# Instruments for measuring the neuromuscular function domain of vitality capacity in older persons: an umbrella review

**DOI:** 10.1007/s41999-024-01017-7

**Published:** 2024-07-08

**Authors:** Francis Louter, Veerle Knoop, Jeroen Demarteau, Ellen Freiberger, Mylene Aubertin-Leheudre, Andrea B. Maier, Jotheeswaran Amuthavalli Thiyagarajan, Ivan Bautmans

**Affiliations:** 1 Frailty & Resilience in Ageing research unit (FRIA), Vitality research group, Vrije Universiteit Brussel, Laarbeeklaan 103, 1090 Brussels, Belgium; 2 Gerontology department, Vrije Universiteit Brussel, Brussels, Belgium; 3 Department of Geriatric Physiotherapy, SOMT University of Physiotherapy, Amersfoort, The Netherlands; 4 Institute for Biomedicine of Aging, FAU Erlangen-Nuremberg, Nuremberg, Germany; 5 Centre de Recherche de l’Institut Universitaire de Gériatrie de Montréal, Montreal, QC Canada; 6 Faculty of Sciences, Department of Exercise Sciences, Université du Québec à Montréal, Montreal, QC Canada; 7 Department of Human Movement Sciences, @AgeAmsterdam, Amsterdam Movement Sciences, Vrije Universiteit Amsterdam, Amsterdam, Netherlands; 8 Department of Medicine and Aged Care, @AgeMelbourne, The University of Melbourne, Melbourne, Australia; 9 The Royal Melbourne Hospital, Parkville, VIC Australia; 10 Yong Loo Lin School of Medicine, Centre for Healthy Longevity, National University of Singapore, Singapore, Singapore; 11 Ageing and Health Unit, Department of Maternal, Newborn, Child and Adolescent Health & Ageing, WHO HQ, Geneva, Switzerland; 12 Geriatrics department, Universitair Ziekenhuis Brussel, Brussels, Belgium

**Keywords:** Intrinsic capacity, Vitality capacity, Hand grip strength, Knee extensor strength, Respiratory muscle strength, Longevity

## Abstract

**Purpose:**

Recently, handgrip, knee extensor and respiratory muscle strength were proposed as candidate biomarkers to assess the neuromuscular function of vitality capacity in older persons. This umbrella review aims to provide an overview of the available instruments and their measurement properties to assess these biomarkers.

**Methods:**

The databases PubMed, Web of Science and Embase were systematically screened for systematic reviews and meta-analyses reporting on handgrip, knee extensor or respiratory muscle strength assessments, resulting in 7,555 articles. The COSMIN checklist was used to appraise psychometric properties and the AMSTAR for assessing methodological quality.

**Results:**

Twenty-seven systematic reviews were included in this study. Some of the identified reviews described the psychometric properties of the assessment tools. We found five assessment tools that can be used to measure neuromuscular function in the context of healthy ageing. Those are the handheld dynamometer for handgrip strength, the dynamometer for knee extensor strength and regarding respiratory muscle strength, the sniff nasal inspiratory pressure, maximal inspiratory pressure (MIP) and maximal expiratory pressure (MEP).

**Conclusion:**

The handheld dynamometer for hand grip strength, the dynamometer for knee extensor strength, sniff nasal inspiratory pressure, MIP and MEP were identified. Therefore, these assessments could be used to identify community-dwelling older adults at risk for a declined neuromuscular function in the context of vitality capacity.

**Supplementary Information:**

The online version contains supplementary material available at 10.1007/s41999-024-01017-7.

## Key summary points

**Aim:**

This umbrella review aimed to identify the available assessments to measure neuromuscular function in community-dwelling older adults, and to critically review their measurement properties.

**Findings:**

Five assessments are suitable for measuring the neuromuscular function domain of vitality capacity in community-dwelling older adults: the handheld dynamometer for hand grip strength, the dynamometer for knee extensor strength, and the sniff nasal inspiratory pressure, maximal inspiratory pressure and maximal expiratory pressure for respiratory muscles.

**Message:**

This study highlights the need and provides evidence for using specific tests for measuring neuromuscular function as a part of vitality capacity in community-dwelling older adults.

**Supplementary Information:**

The online version contains supplementary material available at 10.1007/s41999-024-01017-7.

## Introduction

The World Health Organisation (WHO) advocates to move towards a more positive model of healthy ageing, emphasising an individual’s intrinsic capacity over a disease-focussed approach [1]. Intrinsic capacity encompasses five different domains: locomotion, sensory, cognition, psychological and vitality [2, 3]. Vitality capacity has been suggested as an important domain, since underlying physiological changes can influence other domains of intrinsic capacity [4]. Recently, a new international consensus definition of vitality capacity for healthy longevity was published defining vitality capacity as a “*physiological state (due to normal or accelerated biological ageing processes) resulting from the interaction between multiple physiological systems, reflected in (the level of) energy and metabolism, neuromuscular function, and immune and stress response functions of the body*” [5]. Along this new conceptual definition, potential biomarkers for vitality capacity were identified. The next step is now to develop an operational definition for vitality capacity by identifying those biomarkers for which the best scientific evidence is available. In this umbrella review, we focus on the biomarkers for the neuromuscular attributes of vitality capacity.

Neuromuscular function refers to the interaction of the nervous system and the muscular system that results in isolated muscle contraction. This requires interaction between motor neurons, release of neurotransmitters and activation of muscle fibres [6]. Dysfunction of neuromuscular function might influence other domains of intrinsic capacity [4]. The international expert panel that generated the consensus definition of vitality capacity proposed handgrip strength, knee extensor strength and respiratory muscle strength as excellent candidate biomarkers for neuromuscular function; however, no specific instruments were recommended by the international panel to measure these biomarkers [5]. It has been shown that reduced handgrip strength is strongly related to negative health outcomes such as mortality and disability [7]. Besides, knee extensor strength in older adults is related to other healthy ageing indicators such as functional ability, quality of life [8] and falls [9]. As a result of the age-related decline in muscle strength in the whole body, the respiratory muscle function also decreases [10] which can result in breathlessness that negatively affects daily activities in older adults [11].

The present umbrella review aims to provide an overview of the available instruments and their measurement properties to assess handgrip strength, knee extensor strength and respiratory muscle strength as biomarkers for neuromuscular function in older adults.

## Methodology

### Study protocol and registration

The umbrella review protocol was registered in the international prospective register of systematic reviews (PROSPERO, registration number: 375906). Preferred Reporting Items for Systematic Reviews and Meta-Analyses (PRISMA) guidelines were followed [12].

### Literature search

The databases PubMed, Web of Science and Embase were systematically screened using two different search strings: one for grip strength combined with knee extensor strength (last search 14th February 2023), and another for respiratory muscle strength (last search 6th March 2023). For this review, the following PICO question was used: ‘What are the available screening or assessment tools and their psychometric properties (I) for measuring handgrip strength, knee extensor strength and respiratory muscle strength, as a biomarker of vitality capacity (O) in persons without specific diseases (P)?’. There were no restrictions on the publication date. The full search strategy is presented in Appendix A and included the following keywords: *(risk assessment OR screening OR measurement) AND systematic review*. For grip strength and knee extensor strength those were combined with: *AND (hand strength OR muscle strength dynamometer OR knee extensor strength)*. For respiratory muscle strength the keywords were combined with: *AND (respiratory function tests OR spirometry)*.

**Fig. 1:**
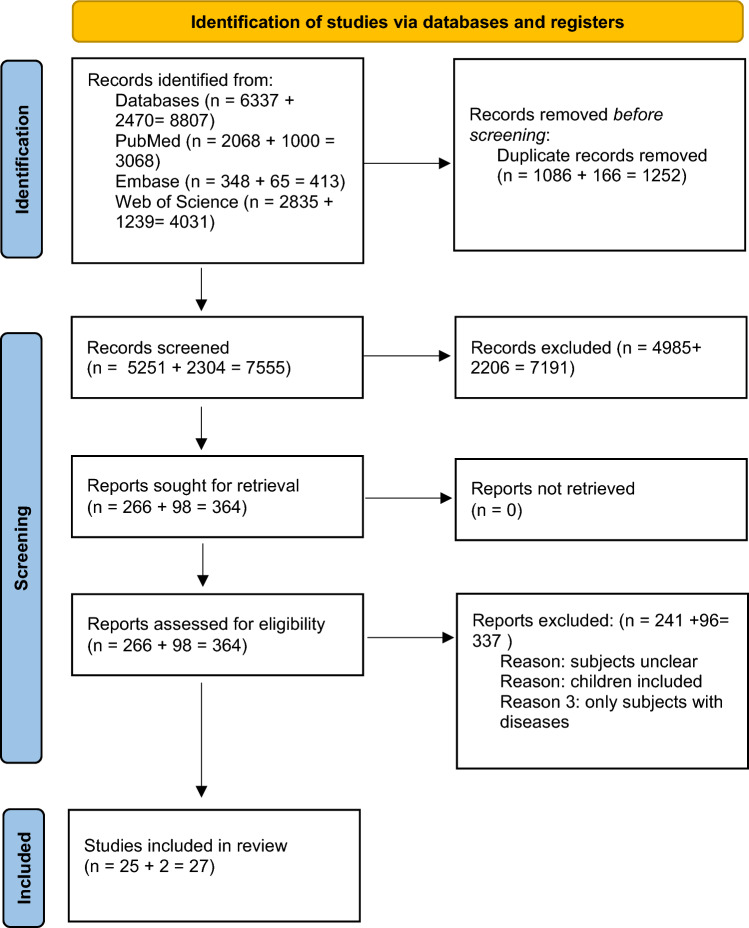
The study selection process

The results were screened independently by two reviewers based on the title and abstract. As a second step, the full text was screened. The reviewers were blind to each other’s decisions. Disagreement between the two reviewers was resolved by discussion and if needed, a third reviewer was consulted. An overview of the selection process can be found in Fig. 1.

### Selection criteria

Studies on assessment tools for neuromuscular function were eligible when they met the following criteria: the studies had to describe instruments for the assessment of handgrip strength, knee extensor strength or respiratory muscle strength, studies had to be systematic reviews or meta-analyses and the participants had to be 18 years or older. Studies that explicitly stated that the sample included participants with (chronic) diseases were excluded; however, studies describing participants at increased risk for disease were not excluded. The studies had to be written in English.

### Data extraction

Two reviewers independently collected data from the selected studies, including the first author, publication year, number of participants, sex, residential status (e.g. community-dwelling), age and the assessment tools used. As a first step, we identified all assessment tools for handgrip strength, knee extensor strength or respiratory muscle strength that were found in the included systematic reviews. However, whilst some of these assessments were described as measures of muscle strength, this characterisation was not consistently applicable across all cases. Therefore, we selected only those instruments that directly measure handgrip strength, knee extensor strength or respiratory muscle strength (as biomarkers of neuromuscular function). Then the psychometric properties of the selected assessment tools and/or the relationship between neuromuscular function and longevity were extracted from the included reviews if this was available. As a last step, the selected assessment tools were discussed by two reviewers and assessed according to criteria for the potential biomarkers, proposed by the international expert panel [5]: (1) feasible to quantify biomarkers or proxy biomarkers, (2) feasible to measure or collect in low-resource setting, (3) useful and informative for monitoring, (4) distinct attribute, (5) acceptable cost and resource demand, (6) sufficient availability and no ethical concern, (7) implementable, and (8) robust psychometric properties. Regarding criterion 8, an extra step was needed in which we used the COSMIN checklist to assess the psychometric properties of the tools that were described [13]. We used the psychometric properties that were described in the included systematic reviews (no additional literature search was performed).

### Quality assessment

The internal validity of the included reviews was assessed using the ‘measurement tool to assess the methodological quality of systematic reviews’ (AMSTAR) [14]. The ‘consensus-based standards for the selection of health status measurement instruments’ (COSMIN) was used to assess the psychometric properties, including validity (criterion, content, structural, predictive, cross-cultural), internal consistency, measurement invariance, reliability (test–retest) and hypothesis testing for construct validity [13]. The COSMIN guidelines were used to evaluate the measurement properties of the assessment tools in terms of adequate ( +), inadequate (−), indeterminate (?) or inconsistent ( ±) based on the study design and methodology. Two reviewers independently rated the AMSTAR and COSMIN. In case of disagreement, consensus was reached after discussion between the reviewers, and if needed, a third reviewer was consulted.

## Results

The literature search resulted in 5,251 articles for the search on hand grip strength and knee extensor strength, and 2,304 articles for the search on respiratory muscle strength. Three hundred sixty-four articles were eligible based on title and abstract. Finally, 27 systematic reviews that were published between 2004 and 2022 were included in this umbrella review (see Fig. 1).

### Quality assessment

The AMSTAR checklist indicated moderate to good methodological quality of the included reviews (see Table 1). No systematic reviews were excluded based on the quality assessment. Only three studies assessed the likelihood for publication bias [15–17].

**Table 1:** Risk of bias assessment

	1. Was an ‘a priori’ design provided?	2. Was there duplicate study selection and data extraction?	3. Was a comprehensive literature search performed?	4. Was the status of publication (i.e. grey literature) used as an inclusion criterion?	5. Was a list of studies (included and excluded) provided?	6. Were the characteristics of the included studies provided?	7. Was the scientific quality of the included studies assessed and documented?	8. Was the scientific quality of the included studies used appropriately in formulating conclusions?	9. Were the methods used to combine the findings of studies appropriate?	10. Was the likelihood of publication bias assessed?	11. Was the conflict of interest included?
Hand grip strength and knee extensor strength											
Moreira et al. (2022) [18]	Y	Y	Y	Y	Y	Y	Y	Y	Y	Y	Y
Kunutsor et al. (2022) [19]	Y	Y	Y	Y	Y	Y	Y	Y	Y	Y	Y
Zasadzka et al. (2021) [20]	Y	Y	Y	Y	Y	Y	Y	Y	Y	Y	Y
Ramsey et al. (2021) [21]	Y	Y	Y	Y	Y	Y	Y	Y	Y	Y	Y
Ramsey et al. (2021) [22]	Y	Y	Y	Y	Y	Y	N	Y	Y	N	Y
de Santana et al. (2021) [23]	Y	Y	Y	Y	Y	Y	Y	Y	Y	Y	Y
Castro-Piñero et al. (2021)[24]	Y	Y	Y	Y	Y	Y	Y	Y	Y	Y	Y
Wang et al. (2020) [25]	Y	Y	Y	Y	Y	Y	Y	Y	Y	Y	Y
Steffl et al. (2020) [26]	Y	Y	Y	Y	Y	Y	N	N	Y	N	Y
Silva et al. (2020)[27]	Y	Y	Y	Y	Y	Y	Y	Y	Y	Y	Y
Dufner et al. (2020) [28]	Y	Y	Y	Y	Y	Y	N	N	Y	N	N
Tarp et al. (2019) [29]	Y	Y	Y	Y	Y	Y	Y	Y	Y	Y	Y
Bergquist et al. (2019) [30]	Y	Y	Y	Y	Y	Y	Y	Y	Y	Y	Y
Kobayashi-Cuya et al. (2018) [31]	Y	Y	Y	Y	Y	Y	N	N	Y	Y	Y
Chamorro et al. (2017) [32]	Y	Y	Y	Y	Y	Y	Y	Y	Y	Y	Y
Dodds et al. (2016) [33]	Y	Y	Y	Y	Y	Y	N	N	Y	Y	Y
Byrne et al. (2016) [34]	Y	Y	Y	Y	Y	Y	Y	Y	Y	Y	Y
Øiestad et al. (2015) [35]	Y	Y	Y	Y	N	Y	Y	Y	Y	Y	Y
Mijnarends et al. (2013) [36]	Y	Y	Y	Y	Y	Y	Y	Y	Y	Y	N
Bohannon et al. (2012) [15]	Y	N	Y	N	N	Y	N	N	N	N	N
Den ouden et al. (2011) [37]	Y	Y	Y	Y	Y	Y	N	N	Y	Y	Y
Cooper et al. (2011) [38]	Y	Y	Y	Y	Y	Y	Y	Y	Y	Y	Y
Cooper et al. (2010) [39]	Y	Y	Y	Y	Y	Y	Y	Y	Y	Y	Y
Bohannon et al. (2008) [7]	Y	Y	Y	Y	Y	Y	N	N	Y	Y	N
Moreland et al. (2004)[9]	Y	Y	Y	Y	Y	Y	Y	Y	Y	Y	N
Respiratory muscle strength											
Souto-Miranda et al. (2021) [40]	Y	Y	Y	Y	Y	Y	Y	Y	Y	Y	N
Janssens et al. (2013) [16]	Y	N	Y	Y	Y	Y	N	N	Y	N	Y

### Characteristics of included studies and instruments

For hand grip strength and knee extensor strength, we included 25 systematic reviews. Table 2 shows the study characteristics and identified assessment tools. The included studies reported on at least 4,704,240 participants, 11 studies did not report the number of participants [7, 9, 15, 20, 26, 30, 32, 34, 36, 38, 39]. Six articles included a ‘healthy’ population [15, 20, 32, 33, 37, 38], and three articles included a specific population: participants with a cognitive risk [19], participants with disability components based on the ICF [18] and one study included participants at risk for diabetes mellitus type 2 [29]. Ten studies included community-dwelling people [18, 20–23, 31, 36–39] and only one study included people living in assisted facilities [36]. Most studies included participants at middle-age (40 years or older) [7, 29, 31] or aged 60 years or older [9, 18, 20–23, 25, 26, 30, 34, 36, 37], though a few also included younger adults (18 years and older) [24, 27, 28]. Almost all studies included men and women, only three studies did not specify [9, 15, 36]. For respiratory muscle strength, we included two systematic reviews, shown in Table 3. One of the included studies reported 9,643 participants [40] and the other study did not report the number of participants [16]. Both of the articles included a ‘healthy’ population [16, 40]. Only one study reported the inclusion of both men and women of 18 years and older [40]. The other study did not report the age and gender of the participants [16].

**Table 2:** Summary of included studies for grip strength and knee extensor strength

Study	Included papers	Participants, age and countries	Assessment tools	Hand grip strength or knee extensor strength	Psychometric properties	Predictive validity on health outcomes
Moreira et al. (2022) ^[[18]]^	6	Age: 60 years and older Participants: average 1030 each paper Gender: men and women Conditions: with disability components based on the ICF Residential status: community dwelling	Hand grip strength: handheld dynamometers: 6 different types are described Knee extensor strength: maximum concentric and eccentric voluntary contraction	Hand grip strength and knee extensor strength	No	Yes Disabilities
Kunutsor et al. (2022) ^[[19]]^	16	Age: not reported Participants: 18,092 Gender: 47.4% males Conditions: general population and at risk of incident cognitive existing cognitive impairment and dementia Residential status: not specified	Handheld dynamometers: 5 different types are described	Hand grip strength	No	Yes Cognitive dysfunction
Zasadzka et al. (2021) ^[[20]]^	16	Age: 60 years and older Participants: not reported Gender: men and women Conditions: without specific diseases Residential status: community dwelling	Handheld dynamometers: 6 different types are described	Hand grip strength	No	Yes Depressive symptoms
Ramsey et al. (2021) ^[[21]]^	21	Age: 60 years and older Participants: 43,796 Gender: 56.4% women Conditions: not specified Residential status: community dwelling Countries: Netherlands	Hand grip strength: handheld dynamometer (different types) Knee extensor strength: Biodex system dynamometer Leg press 1 RM Standard weight stack 5TST 30 s chair stand test 10 chair stand test # chair stand test in 30 s Hydromusculator GT-160 isometric knee extension HF Star Quadriceps strength dynamic contractions against hydraulic resistance Strain Gauge connected to Interface Series SM S-Type Load Cell and Nexus-10 device Lords strap assembly 1 RM KES TTM muscular metre quadriceps strengths # chair stands in 60 s Timed 4.5 chair stand test (ending seated) Microfet Handheld dynamometer knee extensors	Hand grip strength and knee extensor strength	No	Yes Physical activity
Ramsey et al. (2021) ^[[22]]^	112	Age: mean or median age 60 years Participants: sample each paper ranged from 7696 to 43,796 Gender: 39–77% women Conditions: not specified Residential status: community dwelling	Hand grip strength: handheld dynamometry Knee extensor strength: chair stand tests	Hand grip strength and knee extensor strength	No	Yes Physical activity
de Santana et al. (2021) ^[[23]]^	9	Age: 65 years and older Participants: 10,028 Gender: 49% women Conditions: not specified Residential status: community dwelling	Handheld dynamometry	Hand grip strength	No	Yes Mortality
Castro-Piñero et al. (2021) ^[[24]]^	101	Age: 19–64 years old Participants: 10,632 Gender: 52.1% women Conditions: not specified Residential status: not reported	Hand grip strength: handheld dynamometers: 3 different types are described à maximal isometric strength Knee extensor strength: sit to stand test	Hand grip strength and knee extensor strength	Yes Criterion validity	–
Wang et al. (2020) ^[[25]]^	45	Age: 65 years and older Participants: 108,428 Gender: 54.8% female Conditions: not specified Residential status: not reported	Hand grip strength: handheld dynamometer Knee extensor strength: not reported	Hand grip strength and knee extensor strength	No	Yes Activities of daily living
Steffl et al. (2020) ^[[26]]^	19	Age: 65 years and older Participants: not reported Gender: men and women Conditions: not specified Residential status: not reported	Isokinetic dynamometry for knee extension strength: unilateral, bilateral and dominant leg	Knee extensor strength	Yes Values of knee extension in sarcopenia	No
Silva et al. (2020) [27]	21	Age: 18 years and older Participants: 11,101 Gender: men and women Conditions: not specified Residential status: not reported	Hand grip strength: handheld dynamometry Knee extensor strength: Knee extension Chair stand test	Hand grip strength and knee extensor strength	No	Yes Sedentary time and muscular strength
Dufner et al. (2020) [28]	10	Age: 20 years and older Participants: 2,592,714 Gender: men and women Conditions: not specified Residential status: not reported Countries: USA	Dynamometry measured whilst standing, with a straight arm, allowed multiple trials per hand, and used a mechanical handgrip dynamometer adjusted for hand size	Hand grip strength	Yes Trend grip strength	No
Tarp et al. (2019) [29]	22	Age: median cohort baseline age of 45 years Participants: 1,713,468 Gender: men and women Conditions: individuals free of type 2 diabetes at baseline, but with diabetes-associated conditions Residential status: not reported	Maximal hand grip strength Maximal hand grip strength normalised to kilogramme body weight Composite index including multiple muscle groups	Hand grip strength	No	Yes Risk type 2 diabetes
Bergquist et al. (2019) [30]	295	Age: 60–70 years Participants: not reported Gender: men and women Conditions: not specified Residential status: not reported	One repetition maximum hand grip strength Five-time sit to stand One time sit to stand Ten times sit to stand 15 s sit to stand 30 s sit to stand 1-min sit to stand Functional leg extensor strength Single knee extension contractions	Hand grip strength and knee extensor strength	Yes Test–retest reliability and validity	No
Kobayashi-Cuya et al. (2018) [31]	22	Age: 45 years and older Participants: 26,011 Gender: 44.6% men Conditions: not specified Residential status: community dwelling	Handheld dynamometers: 5 different types	Hand grip strength	No	Yes Cognitive function
Chamorro et al. (2017) [32]	30	Age: not reported Participants: not reported Gender: men and women Conditions: asymptomatic participants Residential status: not reported	Handheld dynamometers: 4 different types à isometric or concentric contractions	Knee extensor strength	Yes Reliably and validity	No
Dodds et al. (2016) [33]	60	Age: not reported Participants: 96,537 Gender: men and women Conditions: general population without specific occupational or illnesses Residential status: not reported	Handheld dynamometers: 3 different types	Hand grip strength	Yes Normative data	No
Byrne et al. (2016) [34]	44	Age: 65 years and older Participants: not reported Gender: men and women Conditions: not reported Residential status: not reported	1RM leg press 5 times chair rise	Knee extensor strength	No	Yes Physical performance
Øiestad et al. (2015) ^[[35]]^	11	Age: not reported Participants: 46,819 Gender: men and women Conditions: not specified only knee extensor strength had to be assessed Residential status: not specified	Dynamometry: different types	Knee extensor strength	No	Yes Osteoarthritis
Mijnarends et al. (2013) [36]	62	Age: 60 years and older Participants: not reported Gender: not reported Conditions: not reported Residential status: community-dwelling older people or people in assisted living facilities	Handheld dynamometer Isokinetic dynamometer Leg press Manual muscle testing	Hand grip strength and knee extensor strength	Yes Reliability and validity	No
Bohannon et al. (2012) [15]	5	Age: not reported Participants: not reported Gender: not reported Conditions: healthy individuals Residential status: not reported	Handheld dynamometer	Knee extensor strength	Yes Minimal detectable change	No
Den ouden et al. (2011) [37]	22	Age: older adults, but not specified Participants: 26,614 Gender: 28–100% women Conditions: healthy Residential status: community dwelling	Leg press power Chair stand test Grip strength: not reported	Hand grip strength and knee extensor strength	No	Yes Disability
Cooper et al. (2011) [38]	24	Age: not reported Participants: not reported Gender: men and women Conditions: non-disabled Residential status: community dwelling	Grip strength Chair stand tests	Hand grip strength and knee extensor strength	–	Yes Fractures, cognitive decline, hospitalisation, coronary heart disease
Cooper et al. (2010) [39]	43	Age: mostly older community, but not specified Participants: not reported Gender: men and women Conditions: not reported Residential status: community dwelling	Handheld dynamometer: different types Five times sit to stand test	Hand grip strength and knee extensor strength	Yes	Yes Mortality
Bohannon et al. (2008) [7]	45	Age: middle-aged and older adults, not specified Participants: not reported Gender: men and women Conditions: healthy participants and patients Residential status: not reported	Handheld dynamometers: 13 different types	Hand grip strength	No	Yes Negative health outcomes
Moreland et al. (2004) [9]	13	Age: 65 years and older Participants: not reported Gender: not reported Conditions: not reported Residential status: not reported	Grip strength Knee extensor strength Timed chair stand tests Quadriceps 1RM Knee extension manual muscle testing	Hand grip strength and knee extensor strength	No	Yes Falls

**Table 3:** Summary of included studies for respiratory muscle strength

Study	Included papers	Participants, age and countries	Assessment tools	Psychometric properties	Predictive validity on health outcomes
Souto-Miranda et al. (2021) [40]	20	Age: 18 years and older Participants: 9643 Gender: 5146 women, 4497 men Conditions: healthy participants Residential status: not reported	Pimax and Pemax Gauges calibrated with a pressure transducer Gauges with the range -200 to + 250CMH2O Pressure transducer with a range of + 300CMH2O connected to an amplifier MRP-PC system, mechanical gauge Electronical gauge Ashcroft pressure gauges with a flanged mouthpiece T tube connected to a transductor which was connected to an amplifier Solid-state pressure transducer interfaced with a computer Manual shutter apparatus, using a monometer, aneroid-type gauge Pressure gauge as an integral part of the pneumotachograph Morgan manometer (type Pmax) containing a small leak (internal diameter 2 mm, 2 cm length) connected to a facemask Transportable apparatus connected to a computer System Spirometry system MRP-PC system Calibrated aneroid vacuum manometer Aneroid vacuum manometer Morgan Pmax monitor Pressure gauge A digital manometer Analogical manometer	No International standards	No
Janssens et al. (2013) [16]	77	Age: adults, not specified Participants: not reported Gender: not reported Conditions: Homogeneous samples of healthy participants Residential status: not reported	phrenic nerve stimulation maximal voluntary inspiratory manoeuver (Mueller) powerful sniff manoeuver electromyography	No	No

### Instruments for assessing handgrip strength

Only handheld dynamometers were described for the assessment of handgrip strength. The assessment tools were often described in general and did not specify the type or test protocol. The most reported instruments were hydraulic dynamometers such as JAMAR [7, 19–21, 24, 28, 31, 33, 41, 42], spring dynamometers such as Smedley [7, 18–21, 31] and pneumatic dynamometers such as Martin Vigorimeter [7, 19, 36, 42]. Two studies described that they measured the maximum handgrip strength [24, 29] and only one study described that they normalised the maximum handgrip strength to bodyweight (in kg) [29]. Lastly, one study described the use of a mechanical handgrip dynamometer that was adjusted for hand size [28].

### Instruments for assessing knee extensor strength

For knee extensor strength, the most reported assessment tool was dynamometry [9, 15, 18, 21, 25, 26, 30, 32, 35–37], such as handheld dynamometry and Biodex systems [21]. Also, a hydraulic resistance system such as a leg press was reported to assess knee extensor strength [21, 34, 36, 37]. Different types of chair stand tests based on either the time needed to complete a specific number of repetitions (e.g. 5 repetitions) or the number of repetitions that can be performed within a specific amount of time (e.g. 30 s) were described for measuring knee extensor strength [9, 21, 22, 27, 34, 37, 38]. However, chair stand tests require not only isolated muscle strength but also other functions such as balance and endurance which makes them less suitable to measure neuromuscular function. Lastly, the single knee extension contractions [30] and manual muscle testing were described [9, 36]. However, the single knee extension contraction test is used to measure hamstring muscle length and the manual muscle testing protocol was not clarified, which makes them not suitable to measure neuromuscular function.

### Instruments for assessing respiratory muscle strength

One systematic review reported the maximal inspiratory pressure (MIP) and maximal expiratory pressure (MEP) [40] for assessing the respiratory muscle strength. They described the use of digital manometers, mechanical gauges, a gauge associated with a pneumotachograph and a gauge that was associated with a spirometry system. The assessment protocol varied but most studies performed measurements with the participants in a sitting position whilst using a nose clip. Whilst most studies measured MIP at residual volume and MEP at total lung capacity, also one study was reported that performed both MIP and MEP measurements at different volumes (percentage of vital capacity). The holding pressure varied from ≥ 1 s or > 2 s and the number of repetitions of the procedure varied between 2 and 10, with most studies reporting at least 3 repetitions.

The other study reported the phrenic nerve stimulation, the maximal voluntary inspiratory manoeuvre (Mueller), powerful sniff manoeuvre and electromyography to estimate the respiratory muscle strength [16]. The maximal voluntary inspiratory manoeuvre is referred to as MIP to measure the inspiratory muscle strength and the powerful sniff manoeuvre is commonly described as the sniff nasal inspiratory pressure.

### Psychometric properties

A few included reviews described the psychometric properties of the assessments. For hand grip strength, the Takei dynamometer was found valid and the results for the validity of the DynEx dynamometer were inconclusive [24]. The JAMAR dynamometer was found less accurate than the Takei and DynEx dynamometer for estimating maximal isometric hand grip strength [24]. The handheld dynamometer for assessing knee extensor strength showed a high inter- and intra-rater reliability, as well as a good concurrent and construct validity [36]. Another study also reported a high intra-class correlation coefficient (ICC) of 0.90 and the minimal detectable change (MDC) was highly variable and ranged from 46.0 to 79.0 Newton in older adults [15]. One study investigated the psychometric properties of different chair stand tests. They described the 30 s sit to stand test with an ICC of 0.89 and moderate concurrent validity in community-dwelling adults with a mean age of 70.5 ± 5.5 years [30]. The ICC and construct validity of the 1RM leg press were described as ‘good’ as well [30]. There were no psychometric properties described for the assessments of respiratory muscle strength in the included reviews. The psychometric properties of the identified assessments are shown in Table 4.

**Table 4:** Psychometric properties of the identified assessments

Assessment	N studies	Designed for	Validated population	Reliability	Validity	Responsiveness	Predictive validity on longevity
Handheld dynamometer for hand grip strength	11	Isometric strength hand and forearm muscles	19–65 years old community-dwelling healthy persons	Not reported	Concurrent validity: NR Construct validity: NR Criterion validity: + [24] Content validity: NR (mean difference range − 0.20, *p* > 0.05 to 2.02 kg *p* < 0.001) (*r* = 0.98, *p* < 0.001)	Not reported	Low handgrip strength: worsening ADL and IADL (pooled OR (95% CI) (1.51 (1.34–1.70); 1.59 (1.04–2.31)) [25] Grip strength adjusted for height is associated with mortality [23] The summary hazard ratio for mortality comparing the weakest with the strongest quarter of grip strength was 1.67 (95% CI 1.45 to 1.93) [39] Lower grip strength: increased risk of future fractures, cognitive decline, hospitalisation[38] premature mortality, the development of disability [7], and may be a risk indicator for poor cognitive outcomes such as cognitive impairment, dementia, Alzheimer dementia[19] and depressive symptoms[20] Risk of type 2 diabetes: each standard deviation of higher muscular strength was associated with a 13% lower risk of type 2 diabetes (95% CI 6%–19%) [29]
1RM leg press	4	Knee extensor strength	Not reported	ICC: + [30, 36]	Concurrent validity: NR Construct validity: + [36] Criterion validity: NR Content validity: NR	Not reported	Not reported
5 times sit to stand test	7	Functional knee extensor strength	Not reported	Not reported	Concurrent validity: NR Construct validity: NR Criterion validity: NR Content validity: NR	Not reported	Negative health outcomes [21, 22, 37] and worsening ADL [25] Falls: the combined OR was 1.76 (95% CI 1.31–2.37) for any fall and 3.06 (95% CI 1.86–5.04) for recurrent falls[9]
30 s sit to stand test	7	Functional knee extensor strength	Community-dwelling adults with a mean age of 70.5 ± 5.5 years	ICC: 0.89 + [30]	Concurrent validity: + [30] Construct validity: NR Criterion validity: NR Content validity: NR	Not reported	Falls: the combined OR was 1.76 (95% CI 1.31–2.37) for any fall and 3.06 (95% CI 1.86–5.04) for recurrent falls[9]
BIODEX	1	Knee extensor strength	Not reported	Not reported	Concurrent validity: NR Construct validity: NR Criterion validity: NR Content validity: NR	Not reported	Not reported
Handheld dynamometer knee extensor strength	11	Isometric extensor strength	Middle aged and older adults[43]	ICC: 0.90 + [15, 36] High inter- and intra-rater reliability; ranged from 0.71 to 0.95	Concurrent validity: + [15, 36] Construct validity: + [15, 36] Criterion validity: NR Content validity: NR	The MDC were highly variable and ranged from 46.0 to 79.0 Newton [15]	Falls: the combined OR was 1.76 (95% CI 1.31–2.37) for any fall and 3.06 (95% CI 1.86–5.04) for recurrent falls[9] Disabilities: correlation with age and disabilities, when measured with a hydraulic or digital dynamometer[18] Symptomatic knee osteoarthritis: increased odds for symptomatic knee osteoarthritis in adult women (OR 1.85, 95% CI 1.29 to 2.64) and in adult men (OR 1.43, 95% CI 1.14 to 1.78)[35]
Single knee extension contractions	1	Hamstring muscle length and range of knee extension	Not reported	Not reported	Concurrent validity: NR Construct validity: NR Criterion validity: NR Content validity: NR	Not reported	Not reported
Maximal inspiratory pressure (MIP)	2	A measure of the strength of inspiratory muscles, primarily the diaphragm	Not reported	Not reported	Concurrent validity: NR Construct validity: NR Criterion validity: NR Content validity: NR	Not reported	Not reported
Maximal expiratory pressure (MEP)	1	Maximum positive pressure that can be generated on forced expiration when the abdominal muscles push the diaphragm and the internal intercostals up	Not reported	Not reported	Concurrent validity: NR Construct validity: NR Criterion validity: NR Content validity: NR	Not reported	Not reported
Powerful sniff manoeuvre	1	Inspiratory muscle strength	Not reported	Not reported	Concurrent validity: NR Construct validity: NR Criterion validity: NR Content validity: NR	Not reported	Not reported
Electromyography	1	Based on stimulating the cervical phrenic nerve	Not reported	Not reported	Concurrent validity: NR Construct validity: NR Criterion validity: NR Content validity: NR	Not reported	Not reported

Construct (convergent or divergent) validity is the degree to which scores of a measurement instrument are consistent with the hypothesis. Content validity assesses the degree to which the content of a measurement instrument is an adequate reflection of the construct to be measured. Criterion validity demonstrates the correlation between the measurement of interest and a gold standard. Concurrent validity shows the extent of the agreement between two measures or assessments taken at the same time. [13] Validity was indicated as: + : *r* > 0.70, −:*r* < 0.70. Reliability was indicated as: + : ICC or weighted Kappa > 0.70, −: ICC or weighted Kappa < 0.70

*MID* minimal important difference, *MCID* minimal clinical important difference, *MDC* minimal detectable change, *NR* not reported

### Predictive validity for longevity

Low handgrip strength was associated with both worsening activities of daily living (ADL) and instrumental activities of daily living (IADL) (pooled odds ratio (95% CI) OR = 1.51 (1.34–1.70) and OR = 1.59 (1.04–2.31), respectively) [25]. Handgrip strength measured by hand dynamometry and adjusted for height is associated with mortality in older adults [23]. The summary hazard ratio for mortality comparing the weakest with the strongest quarter of grip strength was HR = 1.67 (95% CI 1.45 to 1.93) [39]. Weaker grip strength was associated with increased risk of future fractures, cognitive decline, hospitalisation [38], premature mortality and the development of disability [7]. Handgrip strength is also associated with the risk of type 2 diabetes: each standard deviation of higher muscular strength was associated with a 13% lower risk of type 2 diabetes (95% CI 6–19) [29]. Besides, handgrip strength may be a risk indicator for poor cognitive outcomes such as cognitive impairment, dementia, Alzheimer dementia [19] and depressive symptoms [20]. Regarding knee extensor strength, it is described that lower body muscle strength and the chair stand test performance were most associated with negative health outcomes [21, 22, 37]. Besides, the chair stand test time was associated with worsening ADL (OR = 1.90 (95% CI 1.63–2.21)) [25]. For lower extremity strength (measured with a dynamometer or a chair stand test), the combined odds ratio was OR = 1.76 (95% CI 1.31–2.37) for any fall and OR = 3.06 (95% CI 1.86–5.04) for recurrent falls [9]. Besides, knee extensor strength correlates significantly with age and disabilities when measured with a hydraulic or digital dynamometer [18]. Also, one systematic review indicated that knee extensor muscle weakness increased the odds of symptomatic knee osteoarthritis in adult women (OR = 1.85, 95% CI 1.29–2.64) and in adult men (OR = 1.43, 95% CI 1.14–1.78) [35]. For the respiratory muscle strength assessments, no information was available.

### Neuromuscular assessments for implementation in vitality capacity

Based on the psychometric properties of the identified tools (see Tables 2 and 3) and the attributes and criteria of vitality capacity (see Table 4) [5], we found five assessment tools adequate for the measurement of neuromuscular function. The handheld dynamometer for hand grip strength and the handheld dynamometer for knee extensor strength were adequate. Regarding respiratory muscle strength, we found the sniff nasal inspiratory pressure, MIP and the MEP potentially adequate (Table 5). However, the psychometric properties of the assessments for respiratory muscle strength remain unclear. The Leg press, BIODEX, and electromyography were excluded from selection due to their impracticality in low-income countries, unacceptable costs, complexity in implementation and insufficient availability. Since chair stand tests do not measure a distinct attribute, these were also excluded from the selection (see Table 5).

**Table 5:** Implementation assessments’ vitality capacity

Neuromuscular function instrument	^b^Feasible to quantify biomarkers or proxy biomarkers	^c^Feasible to measure or collect in low-resource settings	^d^Useful and informative for monitoring	^e^Distinct attribute	^f^Acceptable cost and resource demand	^g^Sufficient availability and no ethical concerns	^h^Implementable	^i^Robust psychometric properties
Handheld dynamometer for hand grip strength^a^	Yes	Yes	Yes	Yes	Yes	Yes	Yes	Yes
1RM leg press	Yes	No	Unclear	Yes	No	No	No	Yes
BIODEX	Yes	No	Yes	Yes	No	No	No	Unclear
Chair stand tests	Yes	Yes	Yes	No	Yes	Yes	Yes	Yes
Handheld dynamometer knee extensor strength^a^	Yes	Yes	Yes	Yes	Yes	Yes	Yes	Yes
Maximal inspiratory pressure (MIP)^a^	Yes	Yes	Unclear	Yes	Yes	Yes	Yes	Unclear
Maximal expiratory pressure (MEP)^a^	Yes	Yes	Unclear	Yes	Yes	Yes	Yes	Unclear
Sniff nasal inspiratory pressure^a^	Yes	Yes	Unclear	Yes	Yes	Yes	Yes	Unclear
Electromyography	No	No	Unclear	Unclear	No	No	No	Unclear

^a^Considered to be adequate to measure neuromuscular function in the context of healthy ageing (based on the implementation assessment)

The scales were graded as: yes, unclear, no

^b^Feasible to quantify biomarkers or proxy markers: not too many items, easy to fill in

^c^Feasible to measure or collect in low- resource settings

^d^Useful and informative for monitoring; responsive to change

^e^Distinct attribute: discriminates cases from non-cases with acceptable level of sensitivity and specificity

^f^Acceptable cost and resource demand

^g^Sufficient availability and no ethical concerns

^h^Implementable, usability: easy to understand, easy to complete

^i^Robust psychometric properties: reliable, reproducibility, accurate

## Discussion

This umbrella review aimed to comprehensively summarise the available literature on assessment tools for neuromuscular function in community-dwelling older adults. The objective was to identify the available and most suitable assessment tools and their psychometric properties. Based on the criteria for vitality capacity, we have identified five assessment tools to measure neuromuscular function. The handheld dynamometer for handgrip strength, handheld dynamometer for knee extensor strength, sniff nasal inspiratory pressure, MIP and the MEP are adequate biomarkers of neuromuscular function within the context of vitality capacity.

We found that handgrip strength was assessed with a handheld dynamometer in all studies and different hydraulic (e.g. JAMAR or SEAHAN systems), pneumatic (e.g. Martin Vigorimeter system) or spring (e.g. Baseline system) dynamometers were described. The handheld dynamometer scored good on all the eight criteria to quantify handgrip strength as a biomarker for vitality capacity. Pneumatic handheld dynamometers, compared to hydraulic handheld dynamometers, have been found to be more sensitive in identifying participants with higher levels of muscle endurance, although both systems can be used [44–47]. The Eforto® system, which consists of a rubber bulb connected to a smartphone-based application, has been found to have good criterion validity and reliability in older community-dwelling persons for (self) measurement of grip strength and muscle fatigability [48]. The use of such assessments holds promise for practical healthcare implementation in the context of vitality capacity. For knee extensor strength, there were different assessment tools found in the literature. Based on the eight criteria for the biomarkers of vitality capacity, we found only the dynamometer for knee extensor strength excellent for assessing the knee extensor strength biomarker. The handheld dynamometer (e.g. Microfet system) showed a high inter- and intra-rater reliability, as well as a good concurrent and construct validity [36]. One study investigated the psychometric properties of different chair stand tests, which described the 30 s sit to stand test with an ICC of 0.89 and moderate concurrent validity in community-dwelling adults with a mean age of 70.5 ± 5.5 years [30]. For the other chair stand tests (e.g. five times sit to stand), the psychometric properties were not reported. However, chair stand tests require not only isolated muscle strength but also other functions such as balance and endurance. Therefore, the chair stand tests are not suitable as assessments to measure the neuromuscular function. Vitality capacity is closely linked with locomotor capacity, one of the other domains of intrinsic capacity. Locomotor capacity is a “state of the musculoskeletal system that encompasses endurance, balance muscle strength, muscle function, muscle power and joint function of the body’’ [49]. Therefore, the chair stand tests are not suitable for assessing vitality capacity but might be useful for assessing the locomotor capacity domain.

For respiratory muscle strength, we found three different assessment tools. Unfortunately, data on the psychometric properties of these tools were scarcely reported in the included review papers. Some systematic reviews reported spirometry as assessment tools for lung function. Spirometry is a test used to measure the ability of a person to inhale and exhale air respective to time, and the forced expiratory volume in one second (FEV1) and forced volume capacity (FVC) are main results of spirometry [50]. A decrease in FEV1 of 10% is associated with a 20% (95%CI 17%–23%) increase in lung cancer risk [51]. Besides, in non-diabetic participants, every 10% decrease in baseline predicted FVC% value was associated with a 13% higher risk of incident diabetes (HR: 1.13, 95% CI: 1.09–1.17) and a similar conclusion could be drawn for FEV1 (HR: 1.10, 95% CI: 1.06–1.14) [52]. However, the spirometry outcomes are useful to categorise the severity of obstructive lung diseases, such as asthma and chronic obstructive pulmonary disease (COPD) [50]. Spirometry does not assess the respiratory muscle strength, which is why the FEV1 and FVC are not eligible as assessments for neuromuscular function. Therefore, we only suggested the use of the MIP, MEP and sniff nasal inspiratory pressure to assess respiratory muscle strength in older adults. Volitional tests such as the MIP and MEP are commonly used to assess the respiratory muscle strength. The MIP measures upper airway pressure during a maximal voluntary inspiratory effort against an occluded mouthpiece and the MEP measures upper airway pressure during a maximal voluntary expiratory effort [53]. Measurement of MIP and MEP can be made with an analogue or digital pressure manometer. The MIP tends to decrease around 40 to 60 years and continues to fall progressively with age, and men tend to have higher MIPs than women [54]. The sniff nasal inspiratory pressure is another non-invasive assessment for the measurement of inspiratory muscle strength. It uses pressure monometers which are inexpensive, and the sniff nasal inspiratory pressure is easy to perform. The digital manovacuometre UFMG has been found to be a reliable and valid instrument for assessing MIP, MEP and sniff nasal inspiratory pressure in adults [55].

The loss of neuromuscular function will lead to a drop of the functional ability of older adults, and an adequate understanding of the underlying mechanisms in skeletal muscle structure is important to understand these age-related changes. Loss of muscle mass is largely due to the progressive loss of motoneurons, which is associated with reduced muscle fibre number and size [56]. The neuromuscular function declines because the motoneuron loss is not compensated by re-innervation of muscle fibres by the remaining motoneurons [6]. At the intracellular level, the ageing process leads to changes in post-translational modifications of muscle proteins and the loss of coordinated control between contractile, mitochondrial and sarcoplasmic reticulum [6]. This loss of muscle strength during the ageing process [57] is a significant contributor of the rising prevalence of frailty and mobility limitations amongst older adults. Notably, functional ability limitations are more pronounced in women compared to men [58]. Women lose muscle mass at a slower rate than men [59] which is intriguing, considering that women experience a higher prevalence of physical disabilities [60]. However, men tend to have a higher muscle mass which protects them during the ageing process since they can lose relatively more muscle mass compared to women. The knee extension peak torque declines 25 years earlier in women, which contributes to the explanation why functional decline occurs more often in women than men [61]. The higher fat mass percentages in women might also contribute to the explanation of the discrepancy in physical functioning between men and women [62]. Interventions that help alleviate muscle mass loss in older adults would be of great benefits in maintaining a better neuromuscular function and reducing the risk of negative health outcomes. Therefore, the assessments as identified in this current umbrella review might allow to identify those persons that are at risk for a declined neuromuscular function and are in need for those interventions to improve their muscle mass.

Vitality capacity has been suggested as an essential domain of intrinsic capacity, since it represents the conditional physiological aspects of intrinsic capacity. A decline in intrinsic capacity amongst older adults is associated with a wide range of adverse outcomes, including impairments in cognitive function, functional ability, sensory perception, physical and mental health, and living standards [63]. Dysfunction of vitality capacity might, therefore, influence various domains of intrinsic capacity, including locomotion, sensory, cognition and psychological capacity. Lower neuromuscular function, higher feelings of fatigue and higher levels of inflammation can negatively influence activities such as walking or raising from a chair [64]. Therefore, a lower vitality capacity can have a negative effect on the locomotor capacity as well. To assess the sensory domain, the most important biomarkers are the hearing and vision abilities. Older people with hearing loss report more disabilities in daily living compared to older people without hearing loss, with older people that contend both hearing and visual problems having the greatest disabilities in daily living [65]. Further research should investigate the relationship of sensory impairment, neuromuscular function and functional declines in daily living. Furthermore, research indicates strong connections between neuromuscular function and cognition, and neuromuscular function is a risk indicator for poor cognitive outcomes such as cognitive impairment, dementia and Alzheimer dementia [19]. In addition, depressive symptoms, recognised as one of the biomarkers of the psychological domain, have been shown to correlate with reduced neuromuscular function as well [20]. Hence, it is essential to adequately assess the vitality capacity domain to gain insights into the intrinsic capacity reserves rather than focussing solely on the vitality capacity domain itself.

This umbrella review has some strength and limitations. We conducted a broad search strategy to identify all assessments for handgrip, knee extension and respiratory muscle strength in the literature. Therefore, the included studies reported on participants that were 18 years and older, even though most studies included older adults. However, the included assessments might not be all validated specifically in older adults, but the results are promising. Furthermore, we have only searched for articles in English, which might have increased the risk of missing relevant research papers. Besides, we did not conduct an additional search for the psychometric characteristics of the assessment tools to prevent any potential bias in the selection process. This decision, however, introduces a limitation to our review, as our discussion on the psychometric properties of these tools is limited to the information provided by the studies that were included in this umbrella review. In particular, we were not able to appraise the psychometric properties of the tools to assess respiratory muscle strength. Moreover, this umbrella review contributes to a better understanding of neuromuscular function assessment in older adults. Whenever we can identify people that are at risk for a decrease in neuromuscular function, we might be able to prevent further decline and functional losses. In doing so, we can encourage healthy ageing by focussing on prevention of adverse health outcomes, rather than treating the clinical manifestations in older adults. Consequently, it is crucial to conduct a validation study of assessments for neuromuscular function and measure the correlation between vitality capacity, healthy ageing and the various domains of intrinsic capacity. Further research could investigate the trajectories of vitality capacity and how to maintain or improve vitality capacity in older adults.

## Conclusion

This umbrella review gives an overview of the available and suitable assessment tools for neuromuscular function for community-dwelling older adults. Five assessments are suitable for measuring the neuromuscular function domain of vitality capacity in community-dwelling older adults: the handheld dynamometer for hand grip strength, dynamometer for knee extensor strength, and the sniff nasal inspiratory pressure, MIP and MEP for respiratory muscles. Further research is necessary to validate these biomarkers and investigate the trajectories of vitality capacity. This study highlights the need of measuring vitality capacity to enhance healthy ageing of community-dwelling older adults. By comprehensively synthesising the available literature and identifying relevant assessment tools, this umbrella review contributes to a better understanding of neuromuscular function assessment in older adults and supports future research and clinical applications.

## Supplementary Information

Below is the link to the electronic supplementary material.

Supplementary file1 (DOCX 22 KB)

## Data Availability

The authors confirm that the data supporting the findings of this study are available within the article and/or its supplementary materials.
