# Supplementary material for: Instruments for measuring the neuromuscular function domain of vitality capacity in older persons: an umbrella review
Source: Eur Geriatr Med. 2024 Jul 8;15(5):1191–213. doi: 10.1007/s41999-024-01017-7 (PMC11614983; doi:10.1007/s41999-024-01017-7)
Supplement: Supplementary file 1 — Supplementary file1 (DOCX 22 KB) [file 41999_2024_1017_MOESM1_ESM.docx]

**Supplementary Material**

For grip strength and knee-extensor strength the following search key was used in PubMed: **(Risk assessment[MeSH Terms]) OR (prognosis[MeSH Terms]) OR (assessment) OR (patient outcome)) OR (tool) OR (test) OR (scale) OR (instrument) OR (screening) OR (measurement) OR (estimation) OR (outcome) OR (predictor) AND (systematic reviews as topic[MeSH Terms]) OR (systematic review[Publication Type]) AND (hand strength[MeSH Terms]) OR (Muscle strength dynamometer[MeSH Terms]) OR (Muscle strength[MeSH Terms]) OR (Hand grip) OR (knee extensor strength) OR (knee extensor power) OR (quadriceps strength) OR (quadriceps power) OR (leg strength) OR (leg extensor power) OR (isokinetic knee extension) OR (neuromuscular function) NOT (COPD) NOT (cystic fibrosis) NOT (cancer) NOT (amyotrophic lateral sclerosis) NOT (Cardiac).**

**The following search key was used for Web of Science and Embase:** (#1 AND #2 AND #3): 1=(**Risk assessment OR prognosis OR assessment OR patient outcome OR tool OR test OR scale OR instrument OR screening OR measurement OR estimation OR outcome OR predictor), 2: (systematic review), 3: (hand strength OR Muscle strength dynamometer OR Muscle strength OR Hand grip OR knee extensor strength OR knee extensor power OR quadriceps strength OR quadriceps power OR leg strength OR leg extensor power OR isokinetic knee extension OR neuromuscular function), NOT COPD NOT cystic fibrosis NOT cancer NOT amyotrophic lateral sclerosis NOT cardiac.**

**For respiratory muscle strength the following search key was used in PubMed: ((((((((((((((((((Risk assessment[MeSH Terms]) OR (Prognosis[MeSH Terms])) OR (assessment)) OR (patient outcome)) OR (tool)) OR (test)) OR (scale)) OR (instrument)) OR (screening)) OR (measurement)) OR (estimation)) OR (outcome)) OR (predictor)) AND ((systematic review[Publication Type]) OR (systematic review))) AND (((((((((((((((respiratory function tests[MeSH Terms]) OR (Maximal respiratory pressures[MeSH Terms])) OR (spirometry[MeSH Terms])) OR (PiMAX)) OR (maximum inspiratory strength)) OR (PeMAX)) OR (maximum expiratory strength)) OR (maximal inspiratory mouth pressure)) OR (maximal expiratory mouth pressure)) OR (sniff nasal)) OR (Sniff manoeuvres)) OR (Diaphragm strength tests)) OR (inspiratory muscle tests)) OR (expiratory muscle tests)) OR (respiratory muscle tests))) NOT (COPD)) NOT (Asthma)) NOT (Cancer)) NOT (Covid-19).**

**The following search key was used for Web of Science and Embase:** (#1 AND #2 AND #3): 1= **Risk assessment OR Prognosis OR assessment OR patient outcome OR tool OR test OR scale OR instrument OR screening OR measurement OR estimation OR outcome OR predictor. 2: (systematic review), 3: (respiratory function tests OR Maximal respiratory pressures OR spirometry OR PiMAX OR maximum inspiratory strength OR PeMAX OR maximum expiratory strength OR maximal inspiratory mouth pressure OR maximal expiratory mouth pressure OR sniff nasal OR Sniff manoeuvres OR Diaphragm strength tests OR inspiratory muscle tests OR expiratory muscle tests OR respiratory muscle tests).**
